# The effects of self-efficacy, social support, and mental toughness on tennis umpires’ professional identity: a study based on a latent variable mediation model

**DOI:** 10.3389/fpsyg.2025.1657181

**Published:** 2025-09-03

**Authors:** Xielin Zhou, Lan Luo, Quandong Liu, Bo Li, Linfeng Wen

**Affiliations:** ^1^Department of Sports Training, Chengdu Sport University, Chengdu, China; ^2^Sichuan Technology and Business University, School of Physical Education and Health, Meishan, China; ^3^Faculty of Education and Humanities, UNITAR International University, Petaling Jaya, Malaysia; ^4^Shanxi University of Chinese Medicine, School of Public Health, Xianyang, China; ^5^Department of Physical Education, Chengdu Sport University, Chengdu, China

**Keywords:** self-efficacy, tennis, umpire, professional identity, social support, mental toughness, chain mediation

## Abstract

**Purpose:**

To explore the relationship between self-efficacy and professional identity among tennis umpires, and to explore the mediating role of mental toughness and social support in this process.

**Methods:**

The Self-Efficacy Scale (GSES), Vocational Identity Scale (VISA), Mental Toughness Scale (CD-RISC10), and Social Support Scale (SSRS) were used to survey 399 tennis umpires in S Province from November 12, 2024, to November 19, 2024.

**Results:**

(1) Correlation analysis showed that self-efficacy and occupational identity showed a significant positive correlation (*r* = 0.544, *p* < 0.01), self-efficacy and social support showed a significant positive correlation (*r* = 0.444, *p* < 0.01), self-efficacy and mental toughness showed a significant positive correlation (*r* = 0.432, *p* < 0.01), social support and mental toughness showed a significant positive correlation (*r* = 0.432, *p* < 0.01), and social support and mental toughness showed a significant positive correlation (*r* = 0.609, *p* < 0.01) (0.432, *p* < 0.01), social support showed a significant positive correlation with occupational identity (*r* = 0.609, *p* < 0.01), and mental toughness showed a significant positive correlation with occupational identity (*r* = 0.504, *p* < 0.01). (2) The results of path analysis showed that self-efficacy significantly and positively affected mental toughness (*β* = 0.165, *p* < 0.01) and social support (*β* = 0.593, *p* < 0.01); mental toughness significantly and positively affected social support (*β* = 0.437, *p* < 0.01); and social support significantly and positively affected occupational identity (β = 0.867, *p* < 0.01). (3) The results of the mediating effect showed that mental toughness partially mediated between self-efficacy and occupational identity, with an effect size of 0.067 and a 95% confidence interval of [0.006, 0.126]; and social support and mental toughness chained the mediating effect between self-efficacy and occupational identity. The effect size was 0.120 with a 95% confidence interval of [0.065, 0.190].

**Conclusion:**

(1) Tennis umpires’ self-efficacy, social support, and mental toughness showed a two-by-two positive correlation with professional identity. (2) Mental toughness plays a simple mediating role between self-efficacy and professional identity, and social support and mental toughness mediate between the two.

## 1 Introduction

The promotion of national fitness activities and mass sports events is of great significance to the development of national fitness. In the context of the national strategy of promoting the construction of a “strong sporting nation,” the construction of a modern, high-level public service system for national fitness is one of the core objectives. The Outline of the Plan for a Strong Sporting Nation (hereinafter referred to as “the Outline”) proposes that “a sports competition system that adapts to the socialist market economy, conforms to the laws of modern sports and conforms to international standards should be established” (China, 2019). The 14th Five-Year Plan for Sports Development further emphasizes the strengthening of the regulation of sports events and the enhancement of competition organization (China, 2021). As the key executors of the competition system, the umpires’ professionalism, professional skills, and state of mental health have a direct impact on the quality of the tournaments, the enthusiasm of public participation and the development of national sports. Among them, the development of tennis is a vivid embodiment of the national strategic layout. Data show that the number of amateur tennis fans in China has reached 23.81 million, an increase of 10.2% compared with 2023 (Network XN, 2024). With the breakthrough of professional tennis in China, amateur tennis is also gradually developing, and the demand for umpires is increasing day by day.

The increase in the scale of tennis tournaments has posed greater challenges to the umpire community. Media reports and feedback from tournament organizers indicate that umpires in mass tennis tournaments face intense work pressure, complex penalty situations and public scrutiny, and that their professionalism (e.g., accuracy in applying the rules) and mental stability (e.g., ability to withstand pressure) on the spot are put to a severe test (Net T, 2024). The frequent occurrence of inappropriate penalties not only affects the fairness of individual matches, but also reduces the public’s motivation to participate in them, thus restricting the healthy development of the national fitness programme (Sohu, 2024). These practical dilemmas suggest that it is significant to explore in depth the drivers of tennis umpires’ professional status.

Focusing on the referee community, Professional Identity is widely recognized as a core psychological foundation for professional behavior and occupational mental health. A high level of professional identity stimulates intrinsic motivation, enhances professional efficacy, increases resilience to stress, and promotes normative professional behavior (Xing Jinming and Jiaqi, 2023). Although the research on professional identity in the fields of teaching and healthcare is relatively mature (Pillen et al., 2013; van Os et al., 2015; Willetts and Clarke, 2014), the association among the formation mechanism, influencing factors, and mental health within the group of sports referees, particularly tennis referees, still requires empirical verification. At the same time, most of the existing research focuses on referring to knowledge and skills or macro-management (Samuel et al., 2017). It constitutes a significant theoretical research gap on the underlying psychological mechanisms, i.e., how referees’ professional identities are shaped, influenced, and interact with their mental health. Based on the self-efficacy theory, social cognitive theory, and professional identity theory, this study takes tennis umpires as an investigative group and probes deeply into the influencing mechanism of their professional identity and its relationship with psychological health. To provide a theoretical basis for the development of the tennis umpire group and empirical support for tennis associations and tournament organizers to select tennis umpires.

## 2 Literature review and research hypothesis

### 2.1 The effect of self-efficacy on professional identity

Professional identity is the core expression of a referee’s internalization of the value of his/her professional role and emotional commitment, which directly affects his/her fairness in officiating, resilience to stress, and sustainability of the profession (Willetts and Clarke, 2014). Bandura’s self-efficacy theory emphasizes that an individual’s beliefs about his or her abilities (i.e., self-efficacy) are the basis for coping with challenges, engaging in occupational roles, and developing a stable identity (Oyserman and Destin, 2010). Previous research has shown that self-efficacy is positively correlated with occupational identity, i.e., as an individual’s self-efficacy increases, his or her occupational identity also increases (Komarraju and Dial, 2014). The underlying mechanism is that individuals with a high sense of self-efficacy are more likely to see challenges at work as opportunities for growth. Self-efficacy is a key factor in regulating interests, goals, and behaviors at work. Individuals with high self-efficacy can positively face occupational challenges, appreciate the meaning and value of work tasks, and develop their occupational identity (Yi et al., 2024). This mechanism is particularly critical in high-pressure occupations, as social work research has shown that low self-efficacy not only hinders the promotion of professional identity but is also significantly associated with high turnover rates (Zhou et al., 2023). Focusing on the group of tennis umpires, their work is characterized by high pressure, the need for instantaneous and precise decision-making, and vulnerability to public scrutiny. Umpires’ beliefs about their ability to make accurate decisions, control the game, and handle disputes (i.e., self-efficacy) are a key psychological resource for their professional identity. Based on this, the present study proposed hypothesis:

*H1:* Self-efficacy positively predicts professional identity.

### 2.2 The mediating role of social support methods

Social support is the psychological support that an individual receives in social interactions, which has a positive effect on alleviating stress reactions, tension, and other negative psychological states (Frisch et al., 2014). Jemini et al. noted a positive correlation between self-efficacy and social support in adolescent populations (Jemini-Gashi et al., 2021). This is in line with Bandura’s ternary interaction theory, which suggests that an individual’s cognitive factors (e.g., self-efficacy) interact with environmental factors (e.g., available social support) (Hu Rui, 2023). Specifically, individuals with high self-efficacy are generally more confident and more inclined to actively seek out and effectively utilize available social support resources to help them solve problems and improve their confidence (Zhang Yue et al., 2023). Also, social support itself is an important environmental factor in shaping self-efficacy (Rippon et al., 2022). For example, affirmation (emotional support) or effective mentoring (instrumental support) from a senior referee can directly increase a novice referee’s confidence in his or her abilities (sense of efficacy).

Secondly, the direct effect theoretical model of social support suggests that social support networks can contribute to positive psychological outcomes such as a sense of purpose, a sense of belonging, and self-identity (Zhou, 2016). According to Maslow’s Hierarchy of Needs theory, when individuals can obtain or feel social support from their classmates, colleagues, or professional peers in their learning and working environments, such support becomes an important source of fulfilment of their belongingness needs and helps them to build a strong sense of identity (Rojas et al., 2023). Relevant studies have shown that social support not only affects teachers’ mental health, but also has a significant impact on their professional identity and job satisfaction (Hu Fangfang, 2013). Kokanovic also proved this idea that social support plays a positive role in the process of individual adaptation and can enhance psychological resilience (Kokanovic et al., 2018). Therefore, this study proposes the hypothesis:

*H2a:* Social support mediates the effect of self-efficacy on the professional identity of tennis umpires.

### 2.3 The mediating role of mental toughness

Psychological resilience is defined as a person’s ability to successfully adapt to or overcome environmental or challenging factors that threaten his or her functioning, survival, and development, and is characterized by its stress-regulating effects as well as its facilitation of adaptation (Gucciardi, 2017). According to Bandura’s social cognitive theory, self-efficacy refers to an individual’s subjective assessment of his or her ability to successfully cope with a variety of challenges from internal and external environments (Kaufmann et al., 2022). This concept influences an individual’s attitudinal tendencies, behavioral choices, level of persistence, and cognitive frameworks etc. Individuals with high self-efficacy tend to demonstrate stronger intrinsic motivation and confidence in themselves, which enables them to adopt positive strategies to cope with adversity and challenges, thus demonstrating greater psychological resilience. Research has shown that individual psychological resilience can enhance research and innovation by increasing self-efficacy (Jinglin, 2024). Experimental research further validates this idea, as Hamann found that in the group of adolescents with insufficient mental toughness, their overall self-efficacy demonstrated significant differences compared to the group with mental toughness through experimental comparisons (Sedillo-Hamann, 2023). This shows that self-efficacy can influence mental toughness.

In addition, it has been found that there is a correlation between mental toughness and career identity (Wu et al., 2022). Individuals with higher mental toughness can demonstrate a greater ability to effectively deal with various challenges in the process of career development, which leads to a more precise and in-depth understanding of their positioning of their professional roles and professional values, and thus significantly enhances their sense of professional identity. Mental toughness can be regarded as a crucial protective element in the process of career development, assisting individuals to maintain a positive mental state in their career paths, and facilitating them to show a high degree of adaptability and flexibility to the ever-changing professional environment. Scholars further pointed out that mental toughness significantly and positively predicted professional identity in a group of primary school physical education teachers, suggesting that mental toughness is an important facilitator of professional identity (Zhao et al., 2022). Erikson’s self-identity theory also proves the point that professional identity belongs to self-identity, and therefore, in constructing professional identity, individuals develop a specific understanding through experience and reflection, which in turn shapes their internal attitudes towards their learned professions and roles, i.e., the construction of self-identity, during which individuals may face psychological conflicts, but some groups can demonstrate strong self-control and flexible adjustment abilities under their psychological resilience and are not easily affected by negative emotions (John et al., 2010; Lundholm et al., 1994). This shows that mental toughness can positively influence occupational identity, therefore, this study proposes the hypothesis:

*H2b:* Mental toughness plays a mediating role in the influence of self-efficacy on the occupational identity of tennis umpires.

### 2.4 Chain mediation of social support and psychological resilience

**Figure 1:**
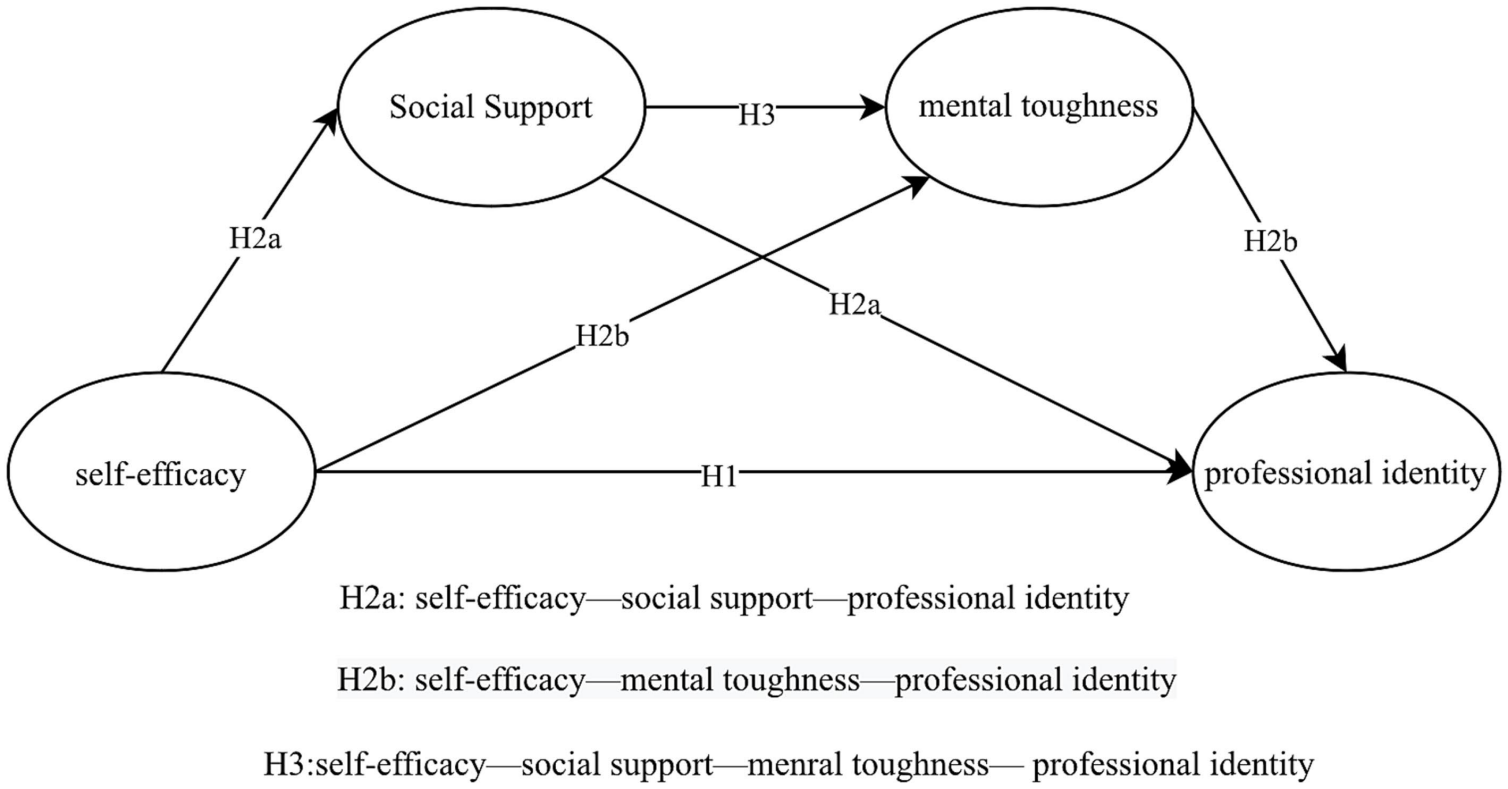
Chain intermediary modeling diagram.

According to Mackinnon, when exploring the relationship between the effects of independent variables on dependent variables in complex scenarios, multiple mediating variables need to be considered to explain the relationship more clearly. Previous studies have shown that self-efficacy, social support, and mental toughness have significant positive effects on career identity and that social support and mental toughness play simple mediating roles, respectively. Meanwhile, psychological resilience refers to an individual’s ability to adapt positively and restore mental health when facing adversity, stress, setbacks, or trauma (Liu et al., 2024), which is influenced by social support, personal cognition, emotion, and other aspects, among which social support is considered an important factor in enhancing psychological resilience (Fan and Lu, 2020). Some scholars have pointed out that social support is significantly positively correlated with mental toughness (Zhikai, 2009). Social support can not only directly affect the level of individual mental health, but also indirectly enhance adaptive capacity by enhancing mental toughness, which is manifested in the fact that the objective support in social support and the individual’s utilization of support can significantly predict mental toughness. Therefore, this study proposes hypothesis:

*H3:* Social support and mental toughness play a chain mediating role in the influence of self-efficacy on the professional identity of tennis umpires (see Figure 1).

## 3 Materials and methods

### 3.1 Participants

As tennis umpires in China are mainly registered members of the provincial tennis associations, daily training, officiating, and assessment information is released through the association’s work group. Therefore, this study used respondent-driven sampling (also known as snowball sampling) to distribute the questionnaires through an online questionnaire programme—Questionnaire Star, where the participants were informed of the content of the questionnaires and agreed to fill them out voluntarily before their distribution, and where the questionnaires were distributed uniformly by the staff of the S Provincial Tennis Association in the workgroups (12/11/2024 to 19/11/2024). The sample size was calculated using the R language program, setting power = 0.80, a Monte Carlo sample size of 20,000 in each repetition, and a confidence level of 95%; the minimum sample size calculated was 126 cases. Based on this, this study distributed 437 questionnaires, 433 were recovered, with a recovery rate of 99.1%, and the samples were excluded based on “repeated answers,” “answer time less than 3 min,” “regular answers,” and “incomplete data.” With “repeated answers,” “answer time less than 3 min,” “regular answers,” and “incomplete data” as the basis for sample exclusion, 399 valid samples were obtained after exclusion, with an effective rate of 92.2%. There are 213 men’s tennis umpires and 186 women’s umpires, with an age range of 21–58 years old, and umpire grades of Grade 3, Grade 2, Grade 1, and National, with final qualifications ranging from specialized to doctoral level (see Table 1).

**Table 1:** Demographic analysis.

Basic information	Form	Frequency	Percentage
Gender	Male	213	53.5
	Female	186	46.5
Age	Under 30 years old	43	10.8
	30–40 years old	156	39.1
	41–50 years old	142	35.6
	Over 51 years old	58	14.5
Referee level	National level	8	2.0
	Level 1 referee	74	18.5
	Level 2 referee	136	34.1
	Level 3 referee	181	45.4
Number of years of refereeing	Less than 5 years	77	19.3
	5 ~ 10 years	74	18.5
	11 ~ 15 years	136	34.1
	More than 16 years	112	28.1
Final degree	Specialist qualifications	41	10.3
	Undergraduate	255	63.9
	Master’s degree	80	20.1
	Doctor’s degree	23	5.8
Add up the total		399	100

### 3.2 Methods

#### 3.2.1 General Self-Efficacy Scale

The scale was developed by Schwarzer and translated by Wang et al. General Self-Efficacy Scale (GSES) (Wang et al., 2001). The GSES is a one-factor scale with 10 items, which uses a 4-point Likert scale, i.e., each item has a rating range from 1 to 4, and participants are asked to respond according to their reality, with higher scores representing higher self-efficacy. In this study, the Cronbach’s alpha coefficient was 0.943, and the model fit showed $\chi^{2}/\mathrm{df}$ = 1.123, GFI = 0.945, CFI = 0.994, IFI = 0.994, TLI = 0.995, and RMSEA = 0.018, indicating that the questionnaire has good reliability and validity.

#### 3.2.2 Social Support Scale

The Social Support Rating Scale (SSRS) was used in this study, which was designed by Xiao Shuiyuan and contains 10 question items divided into three dimensions: objective support, subjective support, and utilization of support (Shuiyuan, 1994). Higher scores on the scale represent greater social support. In this study, the Cronbach’s *α* coefficients of the dimensions of social support were 0.830, 0.673, and 0.841, respectively, and the validation factor analysis was conducted, and the model fitting results showed that $\chi^{2}\mathrm{df}$ =1.171, GFI = 0.980, TLI = 0.997, CFI = 0.998, and RMSEA = 0.021, which indicated that the questionnaire had good reliability and validity.

#### 3.2.3 Mental Toughness Scale

The mental toughness scale used in this study was developed by Drs Campbell-Sills and Stein using factor analysis based on the CD-RISC30. The CD-RISC10 is a one-factor scale with 10 question items scored on a 5-point Likert scale to reflect the overall level of mental toughness of the sample, with higher scores representing greater mental toughness (Campbell-Sills and Stein, 2007). In this study, the Cronbach’s α value of the mental toughness scale was 0.928, a validated factor analysis was conducted, and the model fit showed $\chi^{2}/\mathrm{df}$ = 1.384, GFI = 0.943, CFI = 0.952, IFI = 0.953, TLI = 0.960, and RMSEA = 0.032, which indicated that the questionnaire had good reliability and validity.

#### 3.2.4 Occupational Identity Scale

The scale was compiled by Porfeli et al. The scale is divided into three dimensions: career commitment, career reflection, and career exploration (Porfeli et al., 2011), and consists of 30 question items on a 5-point Likert scale, with higher scores indicating clearer and more stable career identity. In this study, the Cronbach’s alpha coefficients were 0.933, respectively, and a validated factor analysis was conducted, and the model fitting results showed that $\chi^{2}/\mathrm{df}$ =1.418, GFI = 0.978, CFI = 0.991, IFI = 0.991, TLI = 0.987, and RMSEA = 0.033, which indicated that the questionnaire had good reliability and validity.

#### 3.2.5 Data analysis

The data collected in this study were processed and analyzed using SPSS 27.0 and AMOS 26.0. Common method bias test (Harman one-way analysis of variance), descriptive statistics (analysis of demographic variables), correlation analysis (Pearson’s positive correlation coefficient and significance report), independent samples t-test, and analysis of variance (ANOVA) were carried out using SPSS; AMOS was used for the construction of the model and testing of the relationship between the variables, and Bootstrap method was used for the testing of mediating effects. In addition, the model fit of the latent variable mediation model was assessed by $\chi^{2}/\mathrm{df}$ , CFI, GFI, IFI, RMSEA, and other fit indices.

## 4 Results

### 4.1 Common methodology bias test

The Harman one-way test for common method bias was used in this study (Zhou and Long, 2004). The results showed that a total of eight factors with an eigenroot greater than 1 were extracted, of which the first factor cumulatively explained 32.01% of the total variance, which is less than the criterion of 40%, indicating that there is no serious common method bias in this study.

### 4.2 Descriptive statistics and correlation analysis

Descriptive statistics were performed on the demographic variables, which in this study included gender, referee rank, years of officiating, and education. Gender was analyzed using independent samples *t*-test and other demographic variables were analyzed using ANOVA, which showed that there were no significant differences in gender, refereeing grade, and years of officiating for self-efficacy, psychological resilience, social support, and occupational identity (all *p* > 0.05), and there were no significant differences in self-efficacy, social support, and psychological resilience in the dimension of final academic qualification (*p* > 0.05), but for the occupational identity dimension of career commitment, there was a significant difference (*p* < 0.05), where the average score for specialist education was 30.05, for bachelor’s degree and master’s degree was 33.83 and 31.85, respectively, and for doctoral degree was 34.18. The results showed that the level of career commitment of individuals with higher education was generally higher than that of specialists, which statistically demonstrated a significant difference (*F* = 2.809, *p* < 0.05), indicating that academic qualifications have a significant effect on career commitment.

The results of the correlation analysis showed that self-efficacy and professional identity showed a significant positive correlation (*r* = 0.544, *p* < 0.01), self-efficacy and social support showed a significant positive correlation (*r* = 0.444, *p* < 0.01), self-efficacy and mental toughness showed a significant positive correlation (*r* = 0.432, *p* < 0.01), and social support and mental toughness showed a significant positive correlation (*r* = 0.432, *p* < 0.01). social support showed a significant positive correlation with occupational identity (*r* = 0.609, *p* < 0.01), and mental toughness showed a significant positive correlation with occupational identity (*r* = 0.504, *p* < 0.01) (as shown in Table 2).

**Table 2:** Mean, standard deviation and correlation analysis.

Variables	Self-efficacy	Social support	Mental toughness	Professional identity
Self-efficacy	1			
Social support	0.444**	1		
Mental toughness	0.432**	0.432**	1	
Professional identity	0.544**	0.609**	0.504**	1
Mean (M)	32.82	41.03	32.85	98.92
Standard deviation (SD)	9.619	8.751	8.814	22.061

“*” indicates *p* < 0.05, “**” indicates *p* < 0.01.

### 4.3 Latent variable mediation model tests

A latent variable mediation model was constructed through AMOS 26.0, using the method of great likelihood (ML) as the model estimation method, and a chained mediation model of latent variables was constructed with self-efficacy as the independent variable, occupational identity as the dependent variable, and social support and mental toughness as the mediator variables, and the following were selected for the report of the model fit on the suggestion of the study by Jackson et al. (2009) (Table 3).

**Table 3:** Goodness-of-fit indicators for intermediary models.

Fitness index	$\chi^{2}/\mathrm{df}$	RMR	RMSEA	GFI	AGFI	IFI	CFI	TLI
Test value	1.446	0.998	0.033	0.926	0.911	0.978	0.978	0.975

As can be seen in Table 3, among the total model fit indicators, $\chi^{2}/\mathrm{df}$ = 1.446, RMR = 0.998, RMSEA = 0.033, GFI = 0.926, AGFI = 0.911, IFI = 0.978, CFI = 0.978, and TFI = 0.975, all of which were higher than the reference values, indicating a good model fit.

**Figure 2:**
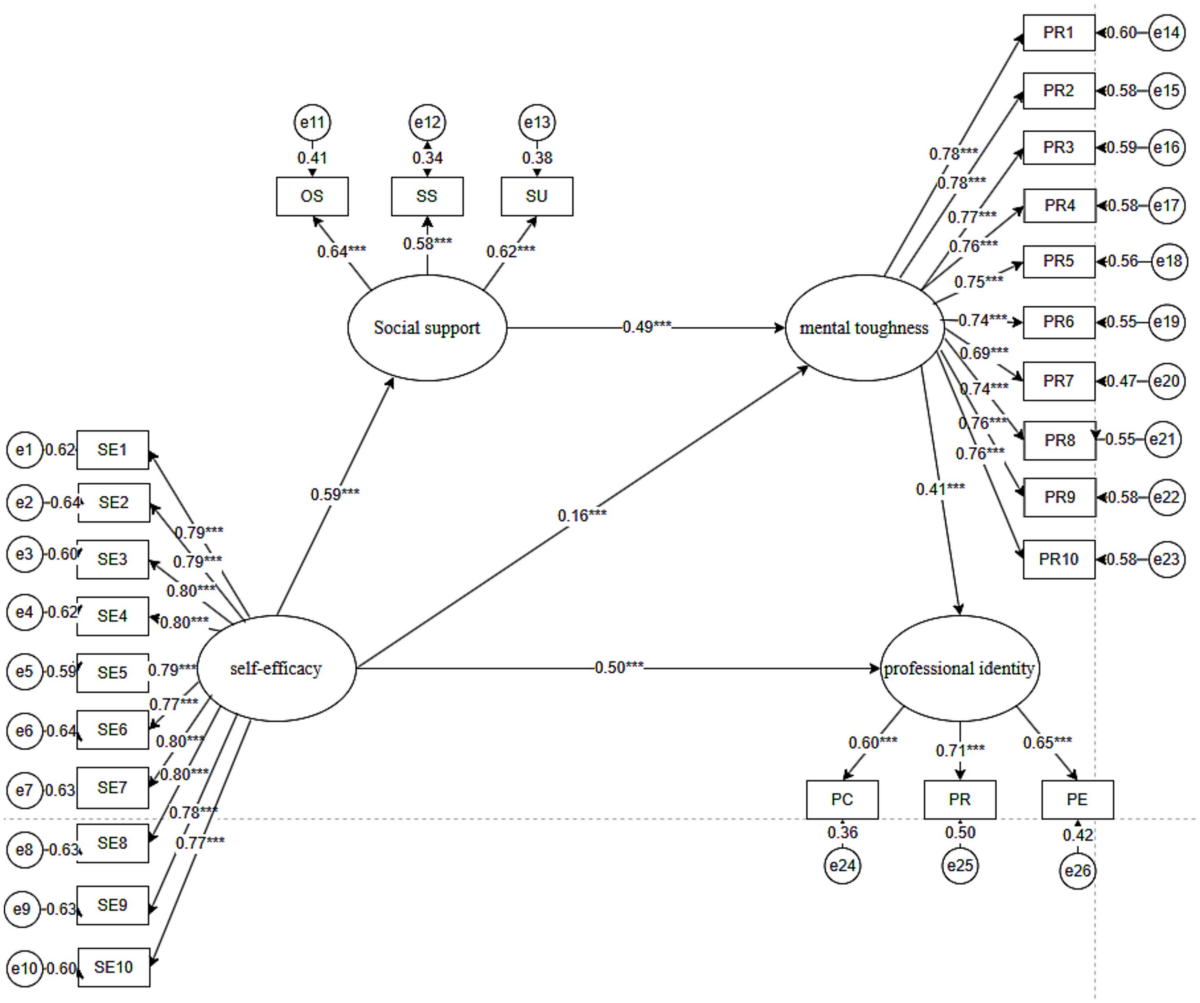
Model path diagram with standardized estimates.

The results of the model are shown in Figure 2, where self-efficacy significantly and positively affects psychological resilience (*β* = 0.165, *p* < 0.01) and social support (β = 0.593, *p* < 0.01). This suggests that individuals with higher self-efficacy typically exhibit higher psychological resilience while being able to access more social support. Social support significantly and positively influenced mental toughness (β = 0.495, *p* < 0.01). This suggests that individuals with higher social support tend to have greater mental toughness. Mental toughness significantly and positively influenced occupational identity (β = 0.409, *p* < 0.01). This suggests that good mental toughness plays an important role in an individual’s occupational identity. The direct effect of social support on occupational identity was not significant. This implies that although social support may indirectly influence occupational identity by enhancing mental toughness, its direct effect has not yet been shown to be statistically significant.

The mediating effect was further tested using the bootstrap method (repeated sampling 2000 times) (Table 4). The results indicated that mental toughness partially mediated the relationship between self-efficacy and professional identity. The effect size was 0.067 with a 95% confidence interval of [0.006, 0.126], not including zero, which indicates that self-efficacy has a partial indirect effect on occupational identity through mental toughness and that this indirect effect is significant. Social support and mental toughness act as chain mediators between self-efficacy and occupational identity. The effect size was 0.120 with a 95% confidence interval of [0.065, 0.190], not including 0. This result suggests that social support and mental toughness together play a chain mediating role, i.e., self-efficacy indirectly influences mental toughness through social support, which in turn influences occupational identity through mental toughness. The indirect effect of this chain effect is equally significant.

**Table 4:** Chained mediation effect test of psychological resilience and social support.

Path relationship	Effect size	Standard error	Bootstrapping 1,000 times 95% CI			
			Bias-corrected		Percentile	
			LLCI	ULCI	LLCI	ULCI
Direct effect	0.496	0.074	0.390	0.599	0.390	0.599
Indirect effect1	0.067	0.030	0.006	0.126	0.003	0.123
chain-mediated effect	0.120	0.031	0.065	0.190	0.066	0.191
Total effect1 (direct effect + mediating effect1)	0.564	0.050	0.469	0.667	0.464	0.661
Total effect 2 (direct effect + chain mediated effect)	0.617	0.052	0.515	0.716	0.516	0.719

## 5 Discussion

### 5.1 Analysis of differences in demographic variables

This study found that gender, umpire rating, years of officiating experience and final academic qualifications of tennis umpires did not show significant differences in psychological traits such as self-efficacy, social support, mental toughness, and professional identity. This finding is different from previous studies, in which Shuhua Wei found significant differences in the sense of identity of practitioners with different levels of experience in her study of professional identity (Wei et al., 2017). Heilman and Chen (2005) also reported gender differences in social support in specific occupational fields, and Mack et al. (2018) also observed a trend of increased mental toughness with increasing professional rank. The reasons for the absence of differences in this study may stem from the specificities of the tennis umpire group: first, the rigorous selection and continuous professional training system (e.g., regular rule updates, case seminars, and on-court assessments) for tennis umpires may have effectively bridged the potential gaps in core psychological traits between umpires of different genders, initial grades, and years of experience, contributing to the development of a relatively homogeneous set of professional psychological qualities. Secondly, the umpire rank itself may reflect more the accumulation of professional skills and experience, such as rule mastery and clinical judgement, than fundamental differences in psychological attributes. Finally, the highly regulated and independent nature of tennis officiating (individual decision-making predominates) may reduce reliance on or sensitivity to specific social identities (e.g., gender, initial academic qualifications), making psychological traits more reliant on an individual’s commitment and adaptation to the profession itself. Together, these factors may lead to a weakening of the influence of the demographic variables on core psychological traits in the specific group of tennis umpires.

However, the final academic qualifications showed significant differences in the career commitment dimension of tennis umpires’ professional identity, specifically, undergraduates and master’s and doctoral students scored significantly higher on career commitment than specialists. According to Social Identity Theory, an individual assigns himself or herself to a particular social group in order to gain a sense of self-identity and self-esteem (Dovidio et al., 2010). Within the field of tennis umpiring, academic qualification, as a key indicator of social identity, profoundly shapes an individual’s perception and judgement of his/her professional identity. Compared with umpires from specialist backgrounds, umpires with undergraduate and doctoral degrees, with their deeper learning backgrounds and broader knowledge reserves, tend to hold a more positive identification with their professional roles and show higher professional loyalty. The root of this difference lies in the fact that, on the one hand, highly educated umpires usually receive richer and more diversified education and training at a deeper level, and have a more thorough understanding of the tennis umpiring profession as well as a higher career vision; on the other hand, higher education tends to open up more career development paths and promotion opportunities for them, which further inspires and consolidates their commitment and dedication to the profession.

### 5.2 The direct effect of self-efficacy on professional identity

This study found that self-efficacy significantly and positively predicted tennis umpires’ occupational identity, with an effect size of 0.496, indicating that hypothesis H1 was valid and consistent with previous research. Based on the viewpoints of self-determination theory, social cognitive theory, and the results of this study, tennis umpires’ self-efficacy can enhance their career confidence and directly improve their career identity. This is demonstrated by the fact that the complexity of career choices is closely linked to an individual’s confidence, and the process of career decision-making is optimized by enhancing self-efficacy, which is therefore seen as a key element in determining an individual’s behavioral intentions and their sustained effort (Xu Cun, 2008). Meanwhile, research in the field of career identity has relied extensively on Super’s theory of career development, highlighting the dynamic nature of changes demonstrated during the developmental stages of a career (Zhang et al., 2023). In summary, this study demonstrated that self-efficacy, as a stable psychological trait, positively predicts tennis umpires’ career identity by integrating self-determination theory, social cognitive theory, and career identity theory.

### 5.3 Analysis of the simple mediating effect of mental toughness

Mental toughness was found to play a simple mediating role in the influence of self-efficacy on the professional identity of tennis umpires, with hypothesis H2b held. Mental toughness, as an intrinsic resource, enhances an individual’s ability to cope positively in the face of challenges and effectively transforms such beliefs into professional identity. For tennis umpires, a high level of self-efficacy inspires a sense of competence and self-confidence in their role, and in addition, mental toughness further consolidates the positive impact of this intrinsic motivation on career identity by enhancing emotional regulation and the ability to cope with stress. This suggests that the role of mental toughness in the process of career development is not limited to regulating emotions and coping with challenges; it can also alleviate the uncertainty and stress faced by referees and provide solid support for their career development. Further, mental toughness enhances referees’ adaptability and decision-making ability in complex game environments, which can become a key node in their career development. However, most of the existing studies have only explored the relationship between self-efficacy and professional identity (Zhou et al., 2023), i.e., self-efficacy can influence other professions’ perceptions of work practices in their field, but have not delved into the mediating role of mental toughness. This study deepened the causal path explanation of the relationship between self-efficacy and occupational identity by introducing the mediating variable of mental toughness, in addition to verifying the role of mental toughness in the specific occupation of tennis referees compared to other occupational groups, which provides a reference for the career development of sports referees and the organization of national fitness events.

### 5.4 Analysis of the chain-mediated effects of social support and psychological resilience

The results of the study indicated that social support and psychological toughness acted as chain mediators prior to self-efficacy and professional identity of tennis umpires, and hypothesis H3 was valid, which is in line with the results of previous studies (Geng et al., 2017). Unlike previous studies, this study does not just consider social support as a direct influence, but further explains how social support indirectly affects occupational identity by enhancing an individual’s internal psychological resources through the introduction of psychological resilience. On the one hand, referees with high levels of self-efficacy can actively seek external support, thereby gaining richer resources to cope with occupational stress. Social support from peers, tournament organizers, and family members provides an important emotional buffer and sense of belonging when referees are faced with player/spectator questioning and even online public pressure for making key calls during a match. On the other hand, the availability of social support enhances referees’ psychological resilience, enabling them to cope more comfortably with professional challenges. This support helps referees to view setbacks as learning opportunities rather than failures (optimism and cognitive restructuring dimensions of psychological resilience), and promotes their rule understanding, clinical judgement, and crisis management skills. Through this transformative learning process of resilience, referees not only improved their professional competence, but also increased their confidence in their own professional competence development and positive outlook on their professional future, which intrinsically drove a more solid professional identity. This finding also further reveals the dynamic process of a referee’s professional identity construction, in which the individual’s internal psychological resources and the social resources in the external environment are intertwined and work together. In addition, building on previous findings (Zhao et al., 2020), the study emphasized that psychological resilience needs to be further affected by professional identity through certain external factors.

However, the results of this study suggest that social support cannot play a simple mediating role in self-efficacy and tennis umpire professional identity, and hypothesis H2a is not valid, which is somewhat different from the results of previous studies. According to the theory of occupational identity, an individual’s sense of identity with an occupation stems from the internal cognition and emotional experience of the occupation’s value, significance, role, and the degree of match between oneself and the occupation (Maloney, 2023). While social support (e.g., encouragement from colleagues, organizational recognition) provides external affirmation and a sense of belonging, the core of occupational identity focuses more on the individual’s understanding of the nature of the occupation, the internalization of values, and the resultant internal commitment to it. Mental toughness is precisely the key trait that underpins an individual’s ability to hold on to professional values, reflect on the meaning of the profession, and deepen inner commitment in the face of setbacks (Quartiroli et al., 2024). Thus, social support may provide more of a favorable external environment or ‘safety net’ for the formation of such deep identities, but its direct impact may not be as direct and powerful as the individual’s intrinsic qualities of cognitive restructuring and resilience (i.e., mental toughness) in shaping the identity itself. Furthermore, within the tennis umpire community, this occupation may rely more on self-evaluation and self-efficacy to shape their professional identity, with social support being relatively less influential. In national fitness tennis events, umpires are required to have a high degree of independence and decision-making ability in the work process, in this scenario, umpires need to rely more on their own experience and internal psychological traits rather than directly through social support to influence their professional identity, therefore, social support fails to directly mediate the process of self-efficacy on the professional identity from tennis umpires, and requires self-efficacy to influence social support, and social support to influence psychological resilience, which in turn affects occupational identity. Therefore, in the future, researchers can further broaden the sample size of the study or introduce other variables (e.g., work-environmental stress) to further observe the potential role of social support in the occupational identity of different groups to enrich the research on the management and development of fitness events for all.

## 6 Conclusion

This study found that, firstly, there was a two-by-two significant positive correlation between self-efficacy, social support, mental toughness, and professional identity. Second, self-efficacy can significantly and positively predict tennis umpires’ occupational identity, which is an important intervening variable influencing tennis umpires’ occupational identity. Third, mental toughness not only plays a simple mediating role in the influence of self-efficacy on tennis umpires’ occupational identity, but self-efficacy can also influence tennis umpires’ occupational identity through the chain mediating role of social support and mental toughness. This mediating effect has important implications for the development of the professional identity of tennis umpires and the promotion of mass tennis tournaments in China, with implications for universal tournaments in other sports.

Although this study explains the relationship between self-efficacy, social support, psychological toughness, and professional identity of tennis umpires, there are some limitations, and future research can address these aspects more thoroughly.

1. This study was cross-sectional and only surveyed tennis umpires in Province S, which is insufficient to cover all regions. Future studies could adopt a longitudinal tracking design to expand the sample size and examine the underlying mechanisms of the variables in both the horizontal and vertical dimensions. For example, tennis umpires from different provinces could be tracked over time to analyze the trends of these variables over time.
2. The use of Respondent Driven Sampling (RDS) in this study (also known as snowball sampling) is inherently non-probability and may introduce a coverage bias, i.e., the sample in this study primarily covers the group of umpires who are managed by, or are closely associated with, the Tennis Association of the Province of S. Referees who are not part of the association community, or who have a weaker connection to the initial “seed” (e.g., independent referees in certain regions, or referees who are not connected to a specific association) are less likely to be included. Therefore, future studies could use stratified random sampling or work with official registry systems to obtain a more comprehensive sampling frame to validate the generalizability of this study’s findings. Also, multi-group comparisons (e.g., different associations, different levels, full-time vs. part-time referees) could be considered to explore the moderating effects of organizational context and individual characteristics on the research model.
3. Due to space constraints, this study only explored the role of social support in the model and did not explore the role of each dimension of social support (objective support, subjective support, and support utilization) dimensionally. Therefore, future research could be designed separately to analyze the separate roles of each dimension of social support in the relationship between self-efficacy, social support, psychological toughness, and professional identity of tennis umpires.

## Data Availability

The original contributions presented in the study are included in the article/Supplementary material, further inquiries can be directed to the corresponding author.
